# Differential effect of canagliflozin, a sodium–glucose cotransporter 2 (SGLT2) inhibitor, on slow and fast skeletal muscles from nondiabetic mice

**DOI:** 10.1042/BCJ20210700

**Published:** 2022-02-11

**Authors:** Hiroko Otsuka, Hisashi Yokomizo, Shintaro Nakamura, Yoshihiro Izumi, Masatomo Takahashi, Sachiko Obara, Motonao Nakao, Yosuke Ikeda, Naoichi Sato, Ryuichi Sakamoto, Yasutaka Miyachi, Takashi Miyazawa, Takeshi Bamba, Yoshihiro Ogawa

**Affiliations:** 1 Department of Medicine and Bioregulatory Science, Graduate School of Medical Sciences, Kyushu University, Fukuoka, Japan; 2 Division of Metabolomics, Medical Institute of Bioregulation, Kyushu University, Fukuoka, Japan; 3 AMED-CREST, Tokyo, Japan

**Keywords:** fast muscle, metabolomics, SGLT2 inhibitor, skeletal muscle, slow muscle

## Abstract

There has been a concern that sodium–glucose cotransporter 2 (SGLT2) inhibitors could reduce skeletal muscle mass and function. Here, we examine the effect of canagliflozin (CANA), an SGLT2 inhibitor, on slow and fast muscles from nondiabetic C57BL/6J mice. In this study, mice were fed with or without CANA under *ad libitum* feeding, and then evaluated for metabolic valuables as well as slow and fast muscle mass and function. We also examined the effect of CANA on gene expressions and metabolites in slow and fast muscles. During SGLT2 inhibition, fast muscle function is increased, as accompanied by increased food intake, whereas slow muscle function is unaffected, although slow and fast muscle mass is maintained. When the amount of food in CANA-treated mice is adjusted to that in vehicle-treated mice, fast muscle mass and function are reduced, but slow muscle was unaffected during SGLT2 inhibition. In metabolome analysis, glycolytic metabolites and ATP are increased in fast muscle, whereas glycolytic metabolites are reduced but ATP is maintained in slow muscle during SGLT2 inhibition. Amino acids and free fatty acids are increased in slow muscle, but unchanged in fast muscle during SGLT2 inhibition. The metabolic effects on slow and fast muscles are exaggerated when food intake is restricted. This study demonstrates the differential effects of an SGLT2 inhibitor on slow and fast muscles independent of impaired glucose metabolism, thereby providing new insights into how they should be used in patients with diabetes, who are at a high risk of sarcopenia.

## Introduction

The skeletal muscle is a major metabolic organ regulating glucose homeostasis through insulin-stimulated glucose uptake and disposal [1,2]. It is composed of four major fiber types based on their contractile properties in adult mammals; a slow-twitch fiber expressing myosin heavy chain (MyHC) I and three fast-twitch fibers expressing MyHC IIa, IIx, and IIb [3]. For example, soleus (SOL), generally classified as slow skeletal muscle (slow muscle), has a high proportion of MyHC I and IIa fibers, whereas extensor digitorum longus (EDL), classified as fast skeletal muscle (fast muscle), has a high proportion of MyHC IIb fibers.

Slow-twitch fibers display a two- to three-fold higher mitochondrial density relative to fast-twitch fibers and are rich in myoglobin and oxidative enzymes, efficient in energy production by fatty acid oxidation, and thus suitable for sustained activities [4], whereas fast-twitch fibers are characterized by glycolytic metabolism and specialized for phasic activities [3,5,6]. Studies with rodents showed that insulin-mediated glucose uptake is higher in slow-twitch oxidative fibers than in fast-twitch glycolytic fibers [7,8]. There is considerable evidence that the proportion of slow-twitch oxidative fibers is decreased in disuse-induced skeletal muscle atrophy, obesity, and diabetes [8–11]. On the other hand, the proportion of fast-twitch glycolytic fibers is known to be decreased by aging and sarcopenia [12]. To date, how slow and fast muscles are differently regulated has not been fully understood [13].

Sodium–glucose cotransporter 2 (SGLT2) inhibitors are an oral antidiabetic drug that promotes urinary excretion of glucose in the renal proximal tubules. The negative energy balance during SGLT2 inhibition leads to the reduction of body weight (BW) and fat mass [14,15]. Given the complex and elaborate organ network originating from the kidney, it is interesting to speculate that SGLT2 inhibitors affect a variety of metabolic pathways in remote organs, outside the kidney. For instance, we have reported that SGLT2 inhibitors; ipragliflozin and canagliflozin (CANA), improve hepatic steatosis in obese mice and delay the onset of nonalcoholic steatohepatitis (NASH)-associated hepatocellular carcinoma in a murine model of human NASH, in association with ‘healthy expansion’ of the adipose tissue [16,17]. On the other hand, there have been clinical and experimental reports on cardio-renal protective effects of SGLT2 inhibitors in rodent models of diabetes and in patients with diabetes [18,19].

There has been a concern that SGLT2 inhibitors could induce skeletal muscle atrophy or sarcopenia [20,21]. As a catabolic response to carbohydrate/calorie loss, SGLT2 inhibitors may induce skeletal muscle degradation to increase the release of amino acids into the systemic circulation, which are transported to the liver as substrates for gluconeogenesis, thereby preventing hypoglycemia. Indeed, luseogliflozin has significantly decreased skeletal muscle index in patients with diabetes [21,22]. On the other hand, tofogliflozin or dapagliflozin has improved glycemic control and reduced BW without reducing muscle mass in patients with diabetes [14,20,23].

SGLT2 inhibitors were originally developed as antidiabetic drugs, and accumulating evidence has supported the use of SGLT2 inhibitors for the treatment of patients with either heart failure or chronic kidney disease, regardless of the presence or absence of diabetes, mainly based on the EMPEROR-reduced trial [24], DAPA-HF trial [25], and DAPA-CKD trial [26]. Based on these evidences, some SGLT2 inhibitors have been globally used for the treatment of nondiabetic patients with heart failure or chronic kidney disease. Additionally, besides diabetes, whether and how SGLT2 inhibition affects slow and fast skeletal muscles remains unclear, independent of impaired glucose metabolism. Therefore, nondiabetic model mice were used in this study to investigate the effect of CANA on the muscle mass and function.

## Results

### Ad libitum feeding experiments (2 weeks)

#### Increased grip strength in CANA-treated mice

**Figure 1.**
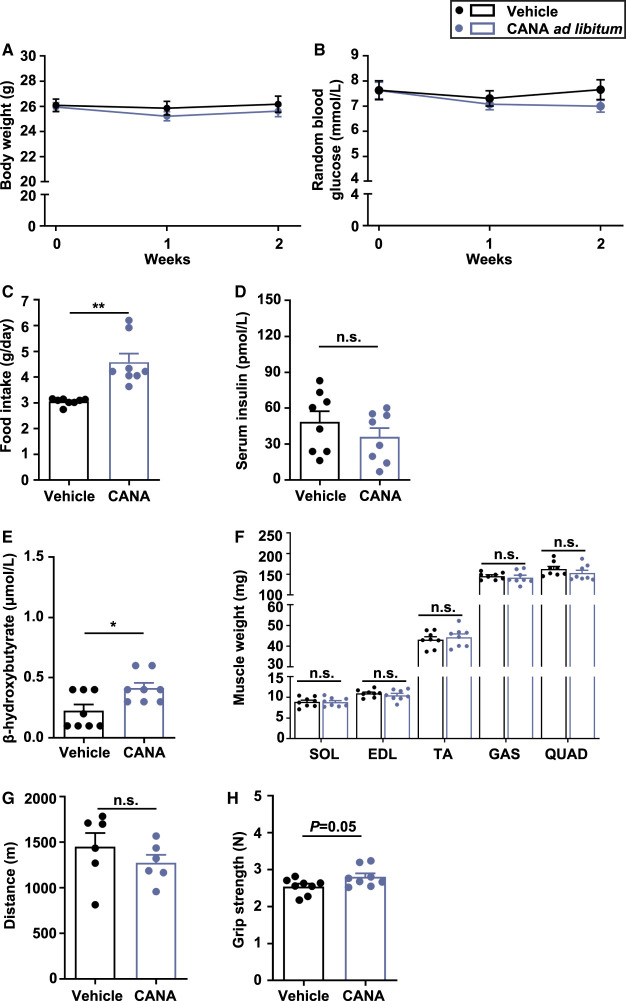
BW, BG, food intake, muscle weight, and function in slow and fast muscles from CANA-treated and vehicle-treated mice during the 2-week *ad libitum* feeding. (**A**,**B**) Change in BW and random BG. (**C**) Food intake. (**D**) Serum insulin after 6-h fasting. (**E**) Blood β-OHB after 6-h fasting. (F) Right lower limb muscle weight. *n *= 8 mice/group. (G) Distance in treadmill exercise. *n *= 6 mice/group. (**H**) Grip strength. *n *= 8 mice/group. Data are expressed as the mean ± SEM. *P < 0.05, **P < 0.01, ***P < 0.001 vs. vehicle-treated mice (Student *t*-test). GAS: gastrocnemius; QUAD: quadriceps; TA: tibialis anterior.

There was no significant difference in BW and random blood glucose (BG) between CANA- and vehicle-treated mice at 2 weeks after the treatment (Figure 1A,B). Food intake was significantly increased in CANA-treated mice relative to vehicle-treated mice (*P* < 0.01) (Figure 1C). There were no significant differences in serum insulin concentration between the two groups (Figure 1D). Blood β-hydroxybutyric acid (β-OHB) was significantly increased in CANA-treated mice (Figure 1E). We found no significant change in slow and fast muscle weight in CANA-treated mice (Figure 1F and Supplementary Figure S1A). There was also no significant difference in liver weight between CANA- and vehicle-treated mice (Supplementary Figure S1B). Epididymal white adipose tissue (eWAT) weight was significantly decreased in CANA-treated mice (Supplementary Figure S1C).

There was no significant difference between CANA- and vehicle-treated mice in terms of running distance, an aerobic and endurance exercise with which to evaluate slow muscle function (Figure 1G). In contrast, grip strength, with which to evaluate fast muscle function, was increased in CANA-treated mice, but no significant difference was observed between the groups (*P* = 0.05) (Figure 1H).

#### Gene expression and metabolome analysis of slow and fast muscles

**Figure 2.**
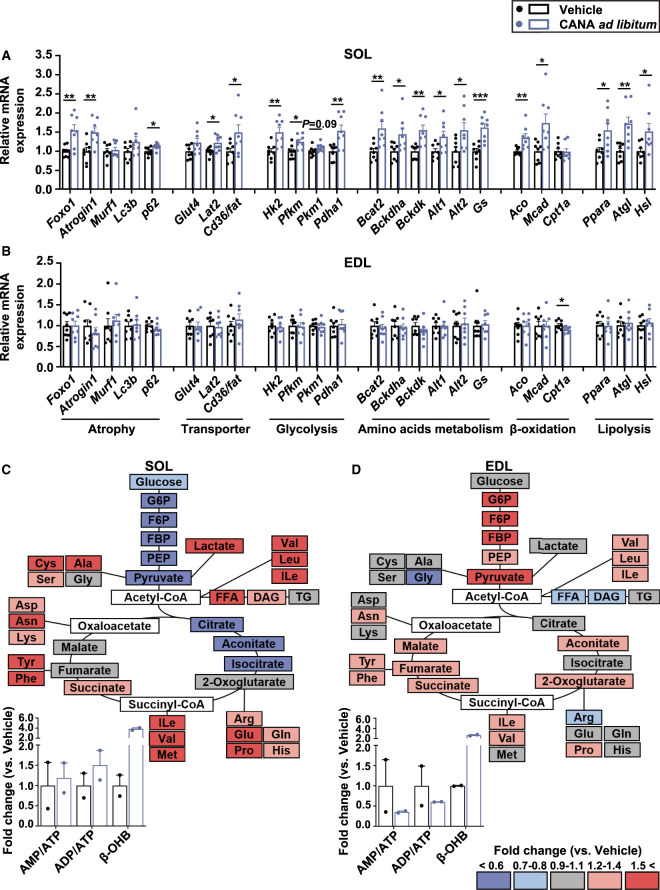
Gene expression and metabolites in slow and fast muscles from CANA-treated and vehicle-treated mice during the 2-week *ad libitum* feeding. (**A**,**B**) The mRNA expression on SOL (**A**) and EDL (**B**). *n *= 6–8 mice/group. (**C**,**D**) The ratio of metabolites in CANA-treated mice to vehicle-treated mice in SOL (**C**) and EDL (**D**), calculated as fold change. Dark red indicates metabolites with the fold change ≧1.5 and dark blue indicates those with the fold change ≦ 0.6. Gray indicates those with no difference between CANA- and vehicle-treated mice. White indicates those undetected. Relative metabolite changes in ATP, ADP, AMP, and β-OHB were shown as bar graphs. White and blue bars represent vehicle- and CANA-treated mice, respectively. *n* = 2 mice/group. Data are expressed as the mean ± SEM. *P < 0.05, **P < 0.01, ***P < 0.001 vs. vehicle-treated mice (Student *t*-test). Ala: alanine; Arg: arginine; Asn: asparagine; Asp: aspartic acid; BCAT2: branched chain amino acid transaminase 2; BCKDHa: branched chain keto acid dehydrogenase E1 subunit α; BCKDK: branched chain ketoacid dehydrogenase kinase; CD36/FAT: fatty acid translocase; CPT1a: carnitine palmitoyltransferase-1a; Cys: cysteine; Gln: glutamine; Glu: glutamic acid; Gly: glycine; His: histidine; Ile: isoleucine; Leu: leucine; Lys: lysine; Met: methionine; Phe: phenylalanine; Pro: proline; Ser: serine; Tyr: tyrosine; Val: valin.

We next examined gene expression in SOL and EDL as slow and fast muscles, respectively, from CANA-treated and vehicle-treated mice (Figure 2A,B). The mRNA expression of muscle atrophy-related genes such as forkhead box transcription factor O1 (*Foxo1*), *Atrogin1*, and *p62* was significantly increased in slow muscle, but unchanged in fast muscle, from CANA-treated mice. The mRNA expression of hexokinase 2 (*Hk2*), which catalyzes the first step in glycolysis by converting glucose into glucose-6-phosphate (G6P), phosphofructokinase, muscle (*Pfkm*), pyruvate kinase muscle isozyme 1 (*Pkm1*), and pyruvate dehydrogenase E1 subunit α 1 (*Pdha1*) were increased in slow muscle, but unchanged in fast muscle from CANA-treated mice. The mRNA expression of amino acid transporter and metabolism genes such as L-type amino acid transporter 2 (*Lat2*), alanine aminotransferase (*Alt1*, *Alt2*), and glutamine synthetase (*Gs*), β-oxidation-related genes such as acyl-CoA oxidase (*Aco*) and acetyl-CoA dehydrogenase, medium chain (*Mcad*), and lipolysis-related genes such as peroxisome proliferator-activated receptor α (*Ppara*), adipose triglyceride lipase (*Atgl*), and hormone-sensitive lipase (*Hsl*) was increased by CANA in slow muscle from CANA-treated mice.

We also examined the effect of CANA on metabolites in slow and fast muscles from two replicates (Figure 2C,D and Supplementary Tables S4 and S5). Overall, amino acids tended to be increased in slow muscle, with no change in fast muscle, from CANA-treated mice relative to vehicle-treated mice. On the other hand, glycolytic metabolites such as G6P, fructose 6-phosphate (F6P), fructose 1,6-bisphosphate (FBP), phosphoenolpyruvic acid (PEP), and pyruvic acid tended to be reduced in slow muscle, and tended to be increased in fast muscle from CANA-treated mice. In CANA-treated mice, free fatty acid (FFA) tended to be increased in slow muscle, but unchanged in fast muscle. Key metabolites in the TCA cycle were mostly unaffected in slow and fast muscles from CANA-treated mice. The ratios of AMP/ATP and ADP/ATP were unaffected in slow muscle, and reduced in fast muscle, from CANA-treated mice. β-OHB tended to be increased in slow and fast muscles, respectively, from CANA-treated mice.

### Pair-feeding experiments (2 weeks)

#### Reduced grip strength in CANA-treated mice

**Figure 3.**
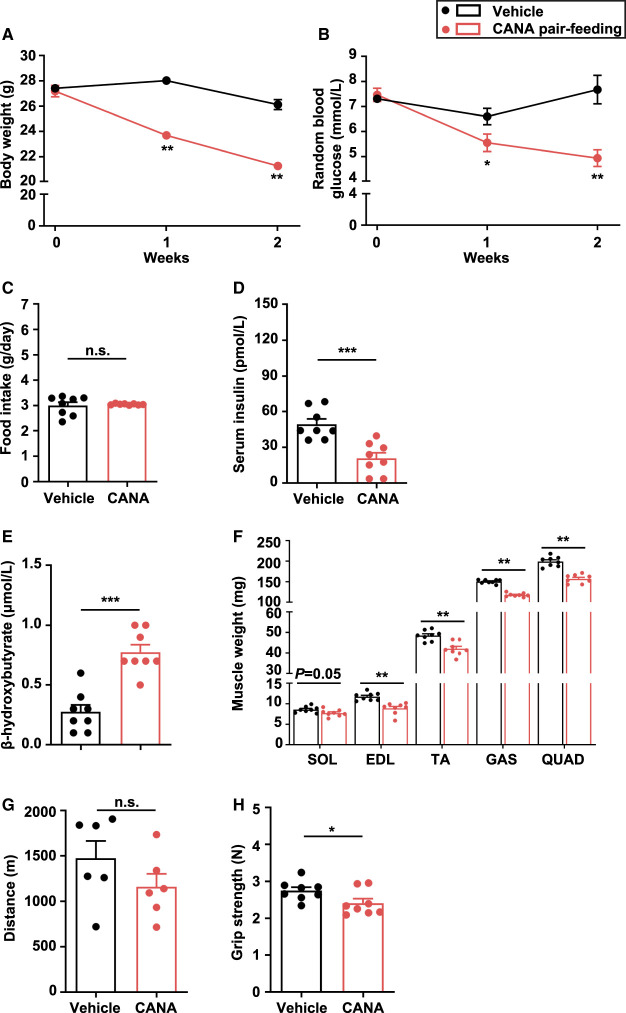
BW, BG, food intake, muscle weight, function in CANA-treated, and vehicle-treated mice during the 2-week pair-feeding. (**A**,**B**) Change in BW and random BG. (**C**) Food intake. (**D**) Serum insulin after 6-h fasting. (**E**) Blood β-OHB after 6-h fasting. (**F**) Right lower limb muscle weight. *n *= 8 mice/group. (**G**) Distance in treadmill exercise. *n *= 6 mice/group. (**H**) Grip strength. *n *= 8 mice/group. Data are expressed as the mean ± SEM. *P < 0.05, **P < 0.01, ***P < 0.001 vs. vehicle-treated mice (Student *t*-test).

Given that increased food intake could affect the metabolic phenotypes in CANA-treated mice, we adjusted the amount of food intake in CANA-treated mice to that in vehicle-treated mice. BW and random BG were significantly reduced in CANA-treated mice relative to vehicle-treated mice during the 2-week pair-feeding (Figure 3A–C). Serum insulin concentration was significantly decreased and blood β-OHB was significantly increased in CANA-treated mice relative to vehicle-treated mice (*P* < 0.05 and *P* < 0.001, respectively) (Figure 3D,E). Slow muscle weight was slightly reduced, but fast muscle weight was significantly reduced in CANA-treated mice (*P* < 0.01) (Figure 3F). However, there was no significant difference in slow and fast muscle weight per BW between CANA- and vehicle-treated mice (Supplementary Figure S2A). The liver and eWAT weight were significantly decreased in CANA-treated mice (Supplementary Figure S2B,C).

There was no difference in running distance between CANA- and vehicle-treated mice (Figure 3G). In contrast, grip strength was significantly reduced in CANA-treated mice relative to vehicle-treated mice (*P* < 0.05) (Figure 3H).

#### Gene expression and metabolome analysis of slow and fast muscles

**Figure 4.**
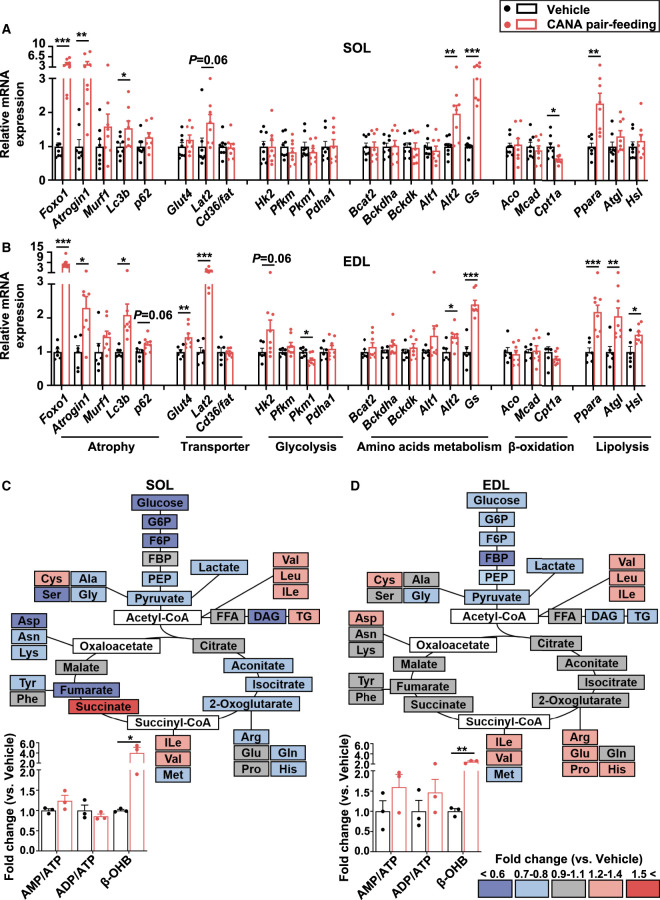
Gene expression and metabolites in slow and fast muscles from CANA-treated and vehicle-treated mice during the 2-week pair-feeding. (**A**,**B**) The mRNA expression on SOL (**A**) and EDL (**B**). *n *= 6–8 mice/group. (**C**,**D**) The ratio of metabolites in CANA-treated mice to vehicle-treated mice in soleus (**C**) and EDL (**D**), calculated as fold change. The color of metabolites in the figure is shown as described above. *n* = 3 mice/group. Data are expressed as the mean ± SEM. *P < 0.05, **P < 0.01, ***P < 0.001 vs. vehicle-treated mice (Student *t*-test).

The mRNA expression of muscle atrophy-related genes such as *Foxo1*, *Atrogin1*, and microtubule-associated protein 1 light chain 3 β (*Lc3b*) was significantly increased in both slow and fast muscles from CANA-treated mice relative to vehicle-treated mice (Figure 4A,B). The mRNA expression of *Glut4* and *Hk2* was unaffected in slow muscle, but increased in fast muscle from CANA-treated mice. The mRNA expression of *Alt2* and *Gs* was significantly increased in both slow and fast muscles. The mRNA expression of lipolysis-related genes such as *Ppara*, *Atgl*, and *Hsl* was increased in fast muscle from CANA-treated mice.

We next examined the effect of CANA treatment on metabolites in slow and fast muscles (Figure 4C,D and Supplementary Tables S6 and S7). Overall, some amino acids were significantly reduced in slow muscle, but unaffected in fast muscle from CANA-treated mice relative to vehicle-treated mice. Glycolytic metabolites were reduced in both slow and fast muscles from CANA-treated mice. FFA and metabolites in the TCA cycle were unaffected in both slow and fast muscles from CANA-treated mice. The ratios of AMP/ATP and ADP/ATP tended to be increased in fast muscle, but unaffected in slow muscle, from CANA-treated mice. β-OHB was significantly increased in slow and fast muscles, respectively, from CANA-treated mice.

### Ad libitum feeding and pair-feeding experiments (4 weeks)

#### Metabolic phenotypes of CANA-treated mice

**Figure 5.**
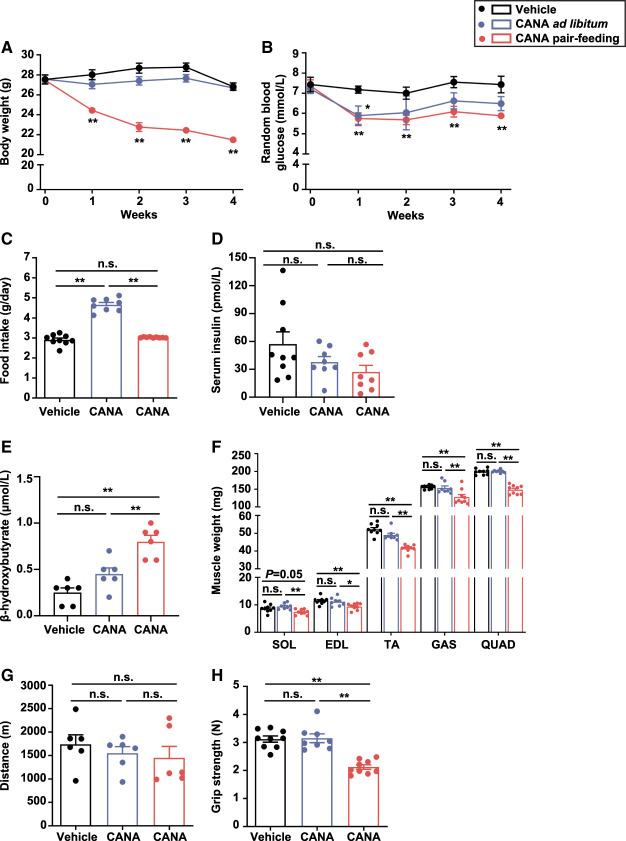
Effect of CANA on BW, BG, food intake, muscle weight, and function. Mice were divided into three groups; those treated with vehicle and those with CANA during the 4-week *ad libitum* feeding, and those treated with CANA during the 4-week pair-feeding. (**A**,**B**) Change in BW and random BG. (**C**) Food intake. (**D**) Serum insulin after 6-h fasting. *n *= 8–9 mice/group. (**E**) Blood β-OHB after 6-h fasting. *n *= 6 mice/group. (**F**) Right lower limb muscle weight. *n* = 8–9 mice/group. (**G**) Distance in treadmill exercise. *n *= 6 mice/group. (**H**) Grip strength. *n *= 8–9 mice/group. White bars are shown for mice treated with vehicle, blue bars for those with CANA during the *ad libitum* feeding, and red bars for those treated with CANA during the pair-feeding. Data are expressed as the mean ± SEM, *P < 0.05, **P < 0.01, ***P < 0.001 vs. vehicle-treated mice (ANOVA).

To investigate the long-term effect of CANA on slow and fast muscles, we extended the duration of CANA treatment up to 4 weeks. Mice were divided into three groups; those treated with vehicle and those with CANA during the *ad libitum* feeding, and those treated with CANA during the 4-week pair-feeding. BW and random BG were unaffected in CANA-treated mice during the *ad libitum* feeding, but decreased during the pair-feeding, relative to vehicle-treated mice (Figure 5A,B). Food intake was significantly increased in CANA-treated mice relative to vehicle-treated mice, when fed *ad libitum* (*P* < 0.01) (Figure 5C). Fasting BG and insulin, and homeostasis model assessment of insulin resistance (HOMA-IR) at 16 h after fasting were not different between CANA-treated mice during the 4-week *ad libitum* feeding and during the 4-week pair-feeding, relative to vehicle-treated mice (Supplementary Figure S3A–C). There was no significant difference in serum insulin concentration among the three groups (Figure 5D). Blood β-OHB was increased in CANA-treated mice during the *ad libitum* feeding with no significant difference, and was significantly increased during the pair-feeding (*P* < 0.01) (Figure 5E). The slow muscle weight was maintained in CANA-treated mice during the *ad libitum* and pair-feeding. On the other hand, fast muscle weight was maintained in CANA-treated mice during the *ad libitum* feeding, but was significantly decreased during the pair-feeding (*P* < 0.01) (Figure 5F). There was no significant difference in slow and fast muscle weight per BW among the three groups (Supplementary Figure S4A). Liver and eWAT weight were maintained in CANA-treated mice during the *ad libitum* feeding, but were significantly decreased during the pair-feeding (Supplementary Figure S4B,C). Running distance was unaffected among the three groups (Figure 5G). Grip strength was unaffected in CANA-treated mice during the *ad libitum* feeding, but significantly decreased in CANA-treated mice during the pair-feeding (*P* < 0.01) (Figure 5H).

The intensity of succinate dehydrogenase (SDH) staining was higher in slow muscle than in fast muscle from vehicle-treated mice. The intensity of SDH staining was unaffected in both slow and fast muscles from CANA-treated mice (Supplementary Figure S4D).

#### The effect of CANA on p-AMPK and p-mTOR expressions in slow and fast muscles

**Figure 6.**
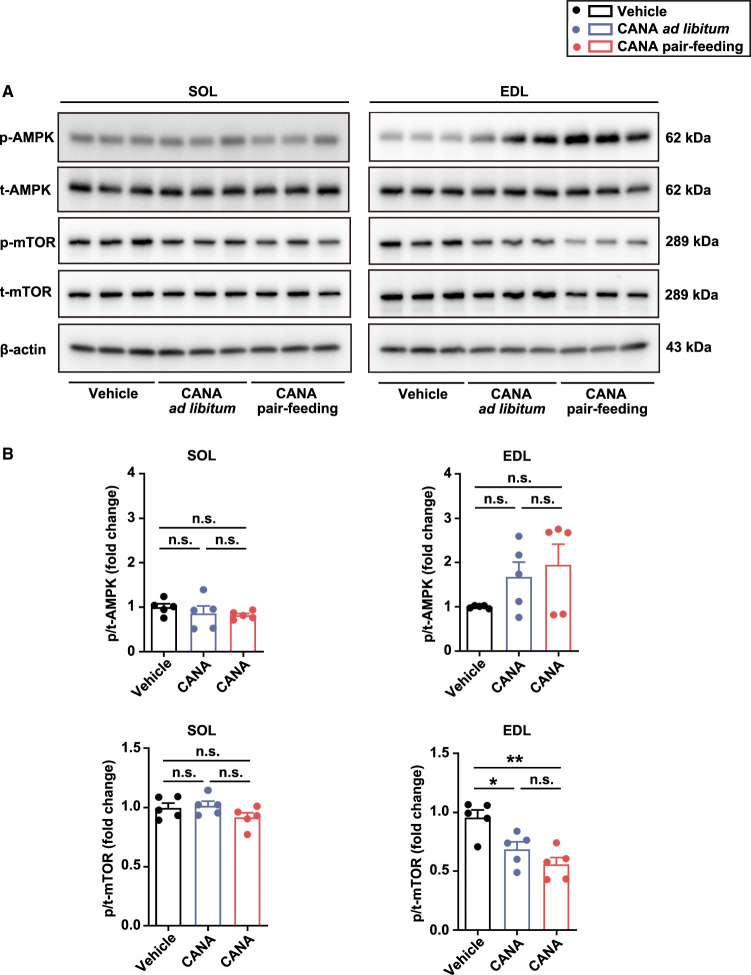
Effect of CANA on the phosphorylated p-AMPK and p-mTOR in SOL and EDL during the 4-week *ad libitum* or pair-feeding. (**A**) Representative Western blots for the assessment of p-AMPK, t-AMPK, p-mTOR, and t-mTOR. (**B**) Quantitative bar graphs. *n* = 5 mice/group. Data are expressed as mean ± SEM. *P < 0.05, **P < 0.01 (ANOVA).

Since it is reported that CANA can increase p-AMPK expression in several cells [27], we performed western blotting and found that the expression of p-AMPK was slightly but not significantly increased in fast muscles from CANA-treated mice during the 4-week *ad libitum* feeding and 4-week pair-feeding relative to vehicle-treated mice, while not affected in slow muscles among the groups (Figure 6A,B and Supplementary Figure S5).

We also found that the expression of p-mTOR was significantly decreased in fast muscles from CANA-treated mice during the 4-week *ad libitum* feeding relative to vehicle-treated mice, and furthermore decreased in fast muscles from CANA-treated mice during the 4-week pair-feeding, while not affected in slow muscles among the groups (Figure 6A,B and Supplementary Figure S5).

#### DNA microarray analysis of slow and fast muscles

Given the marked phenotypic difference between CANA- and vehicle-treated mice during the pair-feeding, we performed microarray analysis of slow and fast muscles obtained from CANA- and vehicle-treated mice during the 4-week pair-feeding. Cluster analysis of gene expression pattern by the pvclust method using the normalized signal data revealed marked difference between slow and fast muscles. We also found marked difference in gene expression pattern in the skeletal muscles between CANA-treated and vehicle-treated mice (Supplementary Figure S6A); 650 and 816 genes were up-regulated in slow and fast muscles, respectively, and 555 and 400 genes were down-regulated in slow and fast muscles, respectively, from CANA-treated mice (Supplementary Figure S6B). Pathway analysis showed that genes related to PI3K–Akt signaling pathway are enriched in both slow and fast muscles from CANA-treated mice (Supplementary Figure S6C). Practically, the gene expression patterns in PI3K–Akt signaling pathways by cluster analysis were quite different between slow and fast muscles as well as vehicle- and CANA-treated mice (Supplementary Figure S7).

#### Gene expression and metabolome analysis of slow and fast muscles

**Figure 7.**
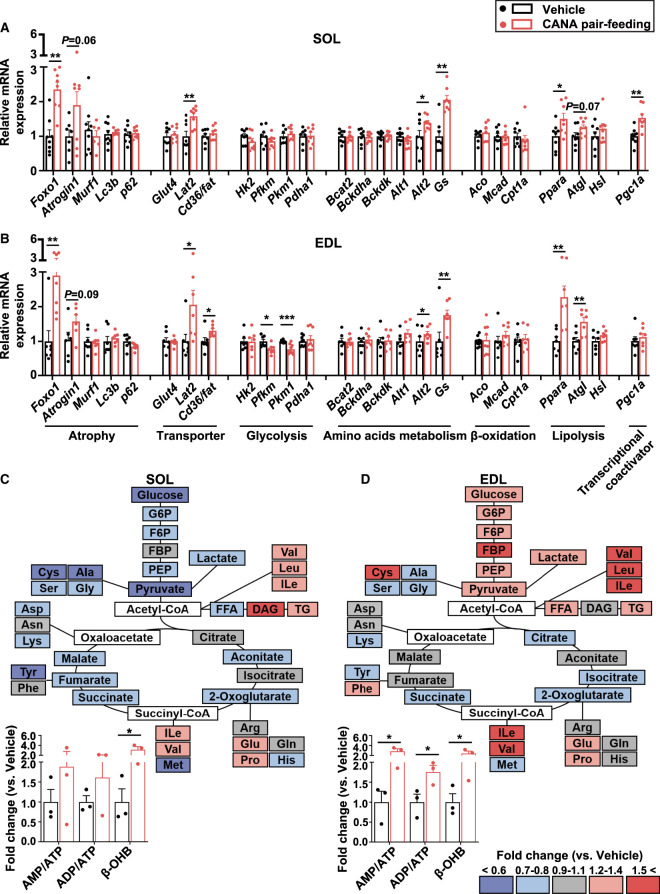
Effect of CANA on gene expression and metabolites in SOL and EDL during the 4-week pair-feeding. (**A**,**B**) The mRNA expression on SOL (**A**) and EDL (**B**). *n *= 6–9 mice/group. (**C**,**D**) The ratio of metabolites in CANA-treated mice to those of vehicle-treated mice in SOL (**C**) and EDL (**D**), calculated as fold change. The colors of metabolites are shown as described above. *n* = 3 mice/group. Data are expressed as means ± SEM, *P < 0.05, ** *P* < 0.01, ***P < 0.001 vs. vehicle-treated mice (Student *t*-test).

The mRNA expression of muscle atrophy-related genes such as *Foxo1* and *Atrogin1* was increased in both slow and fast muscles from CANA-treated mice relative to vehicle-treated mice during the 4-week pair-feeding (Figure 7A,B). The mRNA expression of *Glut4* and glycolytic enzymes *Hk2* were unaffected in slow and fast muscles, whereas that of *Pfkm* and *Pkm1* was significantly reduced in fast muscle but not in slow muscle. The mRNA expression of *Lat2*, *Alt2*, and *Gs* as well as *Ppara* and *Atgl* was increased in both slow and fast muscles from CANA-treated mice. The mRNA expression of peroxisome proliferator-activated receptor γ co-activator 1α (*Pgc1a*), which is involved in the regulation of energy metabolism, mitochondrial biogenesis and increased slow-twitch fibers was significantly increased in slow muscle (*P* < 0.01), but unaffected in fast muscle from CANA-treated mice. Although the mRNA expression of *Pgc1a* was significantly increased in slow muscles from CANA-treated mice, muscle fiber type was unaffected in both slow and fast muscles from CANA-treated mice relative to vehicle-treated mice as determined by immunostaining of MyHC (Supplementary Figure S8).

We next examined the effect of CANA on metabolites in both slow and fast muscles (Figure 7C,D and Supplementary Tables S8 and S9). Overall, some amino acids were significantly reduced in slow muscle, but some amino acids tended to be increased in fast muscle, from CANA-treated mice relative to vehicle-treated mice. Glycolytic metabolites were reduced in slow muscle, but increased in fast muscle, from CANA-treated mice. FFA was reduced in slow muscle, but increased in fast muscle, from CANA-treated mice. Metabolites involved in the TCA cycle were unaffected in slow and fast muscles from CANA-treated mice. The ratios of AMP/ATP and ADP/ATP were unaffected in slow muscle, but significantly increased in fast muscle, from CANA-treated mice (*P* < 0.01). We also found that β-OHB was significantly increased in slow and fast muscles, respectively, from CANA-treated mice.

#### PI3K–Akt pathway activity in slow and fast muscles

We next examined the insulin-stimulated p-Akt levels in slow and fast muscles during the 4-week pair-feeding. Before insulin stimulation, there was no significant difference in p-Akt levels in both slow and fast muscles between CANA-treated and vehicle-treated mice (Supplementary Figure S9A–C). After insulin stimulation, p-Akt levels were increased by 4.3- and 3.2-fold in slow and fast muscles from vehicle-treated mice, respectively. On the other hand, p-Akt levels were increased by 10.9- and 6.5-fold in slow and fast muscles from pair-fed CANA-treated mice, respectively (Supplementary Figure S9A–C).

## Discussion

The skeletal muscle plays a critical role in the regulation of glucose homeostasis through insulin-stimulated glucose uptake and disposal. Because diabetes is often associated with loss of muscle mass and strength or sarcopenia, it is of great importance to understand how a particular antidiabetic drug could affect skeletal muscle mass and function [28,29]. Given that SGLT2 inhibitors induce catabolic responses by reducing glucose reabsorption in the renal proximal tubules [30], there has been a concern about their potential to induce skeletal muscle atrophy. However, the effect of SGLT2 inhibition on the skeletal muscle has not been fully understood [31], and if so, how slow and fast muscles are affected has not been addressed. This study was designed to elucidate the effect of an SGLT2 inhibitor, CANA on the skeletal muscle from nondiabetic mice.

In this study, CANA does not affect slow and fast muscle mass, when mice are fed *ad libitum*. Grip strength is increased, although running capacities are unaffected, suggesting the differential effect of SGLT2 inhibition on slow and fast muscles; fast muscle function appears to be affected preferentially over slow muscle function in mice, when fed *ad libitum*. Importantly, fast muscle mass and function are significantly decreased in CANA-treated mice during the pair-feeding, when slow muscle is unaffected. This is consistent with a previous report by Sano *et al*. [32] that grip strength is increased in patients with diabetes by ipragliflozin, which is accompanied by increased food intake. These observations, taken together, suggest that increased fast muscle function during SGLT2 inhibition is due at least in part to the compensatory increase in food intake.

The regulation of skeletal muscle mass depends upon the balance between protein synthesis and degradation [33–36]. We found that expression of muscle atrophy-related genes is slightly increased in parallel with increased amino acids in slow muscle from CANA-treated mice, when fed *ad libitum*. Since there is no appreciable reduction of slow muscle mass in CANA-treated mice, it is likely that increased amino acids, possibly as a result of increased food intake, are used as substrates for muscle protein synthesis in slow muscle, thus leading to the maintenance of slow muscle mass. The atrophic response is not induced, with no increased amino acids in fast muscle from CANA-treated mice, when fed *ad libitum*. However, during the pair-feeding, muscle atrophy-related genes are induced in both slow and fast muscles from CANA-treated mice. It is, therefore, conceivable that amino acids are used for muscle protein synthesis in CANA-treated mice even during the long-term food restriction, thus leading to the slight decrease in slow muscle mass (Supplementary Figure S10A). On the other hand, glycolytic metabolites are reduced in fast muscle as a result of selective loss of glucose during SGLT2 inhibition, when amino acids are increased by muscle proteolysis to be used as energy substrates, thus leading to significant reduction of fast muscle mass. Interestingly, during the long-term food restriction, glycolytic metabolites are increased with decreased expression of glycolytic genes in fast muscle, suggesting decreased glycolytic flux. Since fast muscle mass is reduced and amino acids are increased by muscle proteolysis, it is likely that amino acids may not be used sufficiently as energy substrates in response to decreased glycolytic flux (Supplementary Figure S10B). Taken together, the mass of both slow and fast muscles is maintained in CANA-treated mice, when fed *ad libitum*, through increased muscle protein synthesis via increased supply of amino acids, when muscle degradation might be slightly induced in catabolic response to SGLT2 inhibition. However, when food intake is restricted with reduced amino acid intake, protein degradation may exceed protein synthesis as a catabolic response to SGLT2 inhibition, which may cause loss of fast muscle predominantly over slow muscle. This discussion is consistent with the notion that muscle proteolysis occurs in fast muscle preferentially over slow muscle during starvation [37]. Previous studies have reported the effect of SGLT2 inhibitors on the mass of skeletal muscle using several mouse models. Treatment with empagliflozin improved the reduction of skeletal muscle mass in Akita mice, which developed diabetes due to pancreatic β cell dysfunction [38]. Also treatment with CANA recovered the reduction of skeletal muscle mass in high-fat diet (HFD)-fed mice, accompanied by increased food intake [39]. As our findings showed that the administration of CANA decreases the skeletal muscle mass during the pair-feeding, but not during the *ad libitum* feeding, further studies will be needed to ask whether SGLT2 inhibitors can affect the skeletal muscle mass in various conditions, including diabetes or HFD-fed state under food restriction.

There is considerable evidence that fast muscle produces ATP mostly via glycolysis, whereas slow muscle does so via the TCA cycle more efficiently [3]. Negative energy balance and reduced blood insulin level during SGLT2 inhibition should induce a variety of catabolic responses outside the skeletal muscles, such as adipose tissue lipolysis, which could contribute to the increased production of FFA and ketone bodies [40]. Evidence has suggested that chronic hyperketonemia in response to SGLT2 inhibition could produce efficient energy via oxidation as a fuel for peripheral tissues, including the skeletal muscle [41]. In this study, ATP production in fast muscle is slightly increased in CANA-treated mice, during the *ad libitum* feeding, but is reduced during the pair-feeding. There is no significant reduction of ATP production in slow muscle from CANA-treated mice even during the pair-feeding. It is, therefore, conceivable that slow muscle is capable of using more substrates such as FFA and β-OHB, and amino acids than fast muscle, to maintain ATP production. In this setting, fast muscle function is increased, but slow muscle function is unaffected in CANA-treated mice, when fed *ad libitum*. However, fast muscle function is reduced in CANA-treated mice during the pair-feeding, when slow muscle function is unaffected. It is likely that CANA maintains ATP production and metabolites from FFA and amino acids (Supplementary Figure S10A), thus maintaining slow muscle function even during the pair-feeding, when glycolytic flux is reduced in fast muscle as a result of reduced glucose supply, thus reducing fast muscle function (Supplementary Figure S10B). Further studies with low carbohydrate or even ketogenic diets are required to elucidate how much glucose is critical in the regulation of slow and fast muscle function during SGLT2 inhibition.

In this study, CANA tended to increase AMPK phosphorylation in fast muscle preferentially over slow muscle, although there is no significant difference. In addition, CANA significantly decreased mTOR phosphorylation in fast muscle, but not in slow muscle. It is likely that the decreased mTOR phosphorylation in fast muscles, but not in slow muscles, led to reduce fast muscle mass in CANA-treated mice during the 4-week pair-feeding. These differential effects of CANA on the phosphorylation of AMPK and mTOR between slow and fast muscle suggest one of the underlying mechanisms by which CANA affects slow and fast muscle mass and function differentially that we observe. Taken together, it is not likely that CANA has a direct effect on muscles, but it is conceivable that CANA affects metabolic pathways in remote organs such as the muscles, by promoting urinary glucose excretion.

The phosphorylation site of Ser^2448^ on mTOR is associated with activation [42] and is phosphorylated in response to Akt activation [43,44]. In subsequent studies, Akt directly phosphorylates TSC1/2 and inhibits its function, subsequently leading to the activation of mTOR [45,46]. In our study, CANA did not affect Akt phosphorylation, whereas CANA reduced Ser^2448^ phosphorylation on mTOR in fast muscle in the fasting state. Considering that AMPK phosphorylates and activates TSC2, thereby inhibiting mTOR activation in response to changes in the intracellular AMP/ATP ratio [47], it is possible that CANA reduced mTOR phosphorylation by slightly increased AMPK phosphorylation, accompanied with elevated AMP/ATP ratio in fast muscle. Since feeding state can affect the phosphorylation of Akt, AMPK, and mTOR, further study will be needed to investigate how CANA affects mTOR phosphorylation in several feeding states.

Regarding the effect of SGLT2 inhibitors on AMPK phosphorylation, there have been some reports on the difference for AMPK phosphorylation among CANA, dapagliflozin, and empagliflozin. It is reported that CANA can increase p-AMPK expression in HEK-293 cells, whereas dapagliflozin and empagliflozin can increase p-AMPK expression with high concentrations [27]. However, oral administration of CANA in mice increased AMPK phosphorylation in liver, but not affected in muscle [27], which is in part consistent with our study. Currently, there have been some reports that not only CANA but also other SGLT2 inhibitors increase AMPK phosphorylation [48]; dapagliflozin increased AMPK phosphorylation in myofibroblasts and in high glucose-treated HK-2 cells [49,50], while empagliflozin increased AMPK phosphorylation in myocardial infarction hearts [51] and improved hepatic steatosis by increasing AMPK phosphorylation [52]. Therefore, SGLT2 inhibitors could increase AMPK phosphorylation by different concentrations in various cells or tissues, although not significantly observed in the skeletal muscles from nondiabetic mice in this study. Taken together, it is conceivable that SGLT2 inhibitors have a class effect on the AMPK phosphorylation. However, there have been few reports on the differential effect of SGLT2 inhibitors, such as dapagliflozin and empagliflozin, on the slow and fast muscle metabolism, including metabolome analysis. Therefore, further study will be needed to ask whether SGLT2 inhibitors have a class effect on exactly the same findings such as muscle metabolism, mass, and function.

The activity of the IGF-1/PI3K/Akt pathway and its downstream targets plays a critical role in the regulation of skeletal muscle mass [53–55]. Evidence suggested that SGLT2 inhibitors improve the skeletal muscle insulin sensitivity [56,57]. In this study, we also found increased insulin-stimulated p-Akt in slow muscle more than in fast muscle from CANA-treated mice during the pair-feeding experiment. This is consistent with a previous report that slow muscle has greater insulin binding capacity, insulin receptor kinase activity, and autophosphorylation relative to fast muscle [58]. Although HOMA-IR was not different among the groups, it is conceivable that CANA can increase insulin sensitivity in the muscles, but without affecting systemic insulin sensitivity in nondiabetic mice. The difference in insulin signaling between slow and fast muscles may result in their differential responses to SGLT2 inhibition.

There are limitations in the current study. (1) We used nondiabetic C57BL/6J mice, which enabled us to assess the effect of SGLT2 inhibition on skeletal muscle independently of impaired glucose metabolism. Considering that diabetes is often associated with increased risk of sarcopenia, leading to physical inactivity, and metabolic disorders especially in older patients [59], and that diabetes is also known to reduce slow muscle mass and function compared with fast muscle [60], further studies with diabetic mice of various etiologies are required to elucidate the differential effect of SGLT2 inhibition on slow and fast muscles in impaired glucose metabolism. (2) We used only male mice. Accumulating evidence has suggested that estrogen plays an important role in the muscle function [61]. Importantly, loss of estrogenic function contributes to alter muscle function and increases the risk of various chronic diseases including sarcopenia, type 2 diabetes, metabolic syndrome, and cardiovascular disease [62]. To exclude the direct effect of estrogen on muscle function, only male mice were used in this study. (3) Metabolome analysis during the 2-week *ad libitum* feeding was obtained from two replicates. Therefore, increased number of samples in metabolome analysis will be needed for better understanding of this study. (4) We measured several gene expressions including ATGL, HSL, and GLUT4, but enzyme activities are not measured for the understanding of the mechanism, indicating the limitation for the interpretation of this study.

In conclusion, this study demonstrates the differential effect of SGLT2 inhibition on slow and fast muscles, which is affected by the amount of food intake. The fiber type-specific effect of SGLT2 inhibition might be explained by the fiber type-specific metabolic properties; during SGLT2 inhibition, fast muscle, where glycolytic metabolites are used to produce ATP, is more affected as a result of selective loss of glucose, than slow muscle, where substrates besides glucose, such as FFA and amino acids, can be used to produce ATP via fatty acid β-oxidation. The data of this study provide insights into how SGLT2 inhibitors should be used for the treatment of patients with diabetes, who are at a high risk of sarcopenia.

## Materials and methods

### Animals and experimental protocols

Eleven-week-old C57BL/6J male mice were purchased from Charles River Laboratories Japan Inc. (Yokohama, Japan). The animals were housed in a temperature-, humidity-, and light-controlled room (12-h light and 12-h dark cycle) and allowed free access to water and normal chow diet (ORIENTAL YEAST CO., LTD. Tokyo, Japan) at Research Center for Human Disease Modeling, Graduate School of Medical Sciences, Kyushu University (Fukuoka, Japan). The animal room was kept at 23 °C during the experiments. CANA was provided by Mitsubishi Tanabe Pharma Corporation, Medicinal Chemistry Laboratory (Saitama, Japan). Twelve-week-old C57BL/6J male mice were fed *ad libitum* with or without 0.03% (w/w) CANA for 2 or 4 weeks. The dose of CANA was set at 30 mg/kg, equivalent to 0.03% (w/w), which has been shown to be an effective pharmacological dose in C57BL/6J mice [63]. The duration of CANA treatment for mice were determined based on the previous study where 4-week administration of 30 mg/kg CANA reduced BW and BG levels in C57BL/6J mice [64]. Food intake was monitored for 3 consecutive days at each week by weighing the mouse feeder MF-4S (SHIN FACTORY, Fukuoka, Japan), and the average daily food intake was calculated. In the pair-feeding experiment, 12-week-old mice were fed with 3.0 g/day of normal chow diet with or without 0.03% (w/w) CANA for 2 or 4 weeks. BW and random BG were monitored once a week during the experiments.

At the end of the experiments, mice were anesthetized with isopentane, and blood samples were obtained from the inferior vein. The weight of the muscles, liver, and eWAT was measured. The slice through the entire midbelly of SOL, EDL, and tibialis anterior (TA) was mounted on cork in optimal cutting temperature, compounded, and frozen in cooled liquid isopentane. Samples were stored at −80°C until use. SOL was examined as slow muscle, while EDL and TA were examined as fast muscles. All experimental protocols were reviewed and approved by the Committee on the Ethics of Animal Experiments, Kyushu University (Protocol #A19-117-4). All methods involving animals were performed in accordance with the relevant guidelines and regulations.

### Assays for biochemical parameters

BG levels were measured using the Stat Strip XP3 (NIPRO, Osaka, Japan), which is a glucose oxidase-based glucose meter. Serum insulin concentrations were measured using enzyme-linked immunosorbent assays (ELISA) (Morinaga Institute of Biological Science, Yokohama, Japan). Blood β-hydroxybutyric acid (β-OHB) was measured using an automatic ketometer (Nipro, Osaka, Japan).

### Grip strength measurement and treadmill exercise

Grip strength of mice was measured with MK-380CM/R (Muromachi Kikai, Tokyo, Japan). After the forelimbs and hindlimbs were placed on a mesh, mice were gently pulled back until it lost its grip from the mesh. The maximal force generated at the point where the animal loses its grip was measured. The highest score among five trials with 30-s recovery period was recorded.

Treadmill exercise test was performed as previously described [65] using Ratbelt-2000 (ARCO SYSTEM, Chiba, Japan). All mice were acclimated 4–5 days prior to the exercise test session. After acclimation, they were allowed to rest for at least 2 days. The exercise test session began at a rate of 12 m/min for 40 min, and thereafter, the speed was increased at the rate of 1 m/min every 10 min for a total of 30 min, and then increased at the rate of 1 m/min every 5 min until they were exhausted (mice spent more than 5 s on the electric shocker without resuming running). The running time was measured and the running distance was calculated.

### Quantitative real-time PCR analysis

Total RNA was extracted from the muscle and cDNA was synthesized as described previously [66]. The PCR primers used are listed in Supplementary Table S1. mRNA levels were normalized to those of 18S mRNA.

### Determination of mitochondrial content

Sections from the muscles were stained for SDH (complex II of the respiratory chain), reflecting oxidative metabolism as previously described [67]. Sections were captured on BZ-8000 microscope (Keyence, Osaka, Japan). For mitochondrial content determination, the mean gray intensity for sections were measured using BZ-II Analyzer (Keyence), allowing the evaluation of mitochondrial content.

### Determination of fiber type

The sections of each muscle were immunolabeled for different MyHC as previously described [68]. Briefly, the cross-sections were used to be immunolabeled for MyHC type I, IIa, and IIb. They were allowed to reach room temperature and rehydrated with PBS (pH 7.2). These sections were then blocked by goat serum (10% in PBS) and incubated for 1 h at room temperature with the primary antibody cocktail. The muscle cross-sections were then washed three times in PBS before incubation for 1 h at room temperature with the secondary antibody cocktail. Cross-sections were then washed three times in PBS and slides were cover-slipped by VECTASHIELD Hard Set Mounting Medium (Vector Laboratories, H-1400). All primary antibodies for MyHC were purchased from the Developmental Studies Hybridoma Bank (DSHB, University of Iowa, IA). Antibodies used are listed in Supplementary Table S2.

### Western blotting

Fresh mouse muscles were homogenized in the RIPA buffer containing protein inhibitor (Nacalai tesque, Kyoto, Japan) and a phosphatase inhibitor (PhosSTOP, Roche, Switzerland), prior to SDS–PAGE. Protein bands were visualized using the ECL Western Blotting Detection System (GE Healthcare, Buckinghamshire, U.K.) and bands densities were assessed by Image J (NIH). The phosphorylation of AMPK (p-AMPK), mTOR (p-mTOR), and Akt (p-Akt) was evaluated by collecting SOL and EDL after 6 h of fasting. In addition, Akt phosphorylation by insulin stimulation was evaluated by administering 0.5 U/kg of insulin from the inferior vena cava and collecting SOL and EDL 10 min later. Antibodies used are listed in Supplementary Table S3.

### Metabolome analysis

Metabolite extraction was performed from the crushed frozen skeletal muscle using the Bligh and Dyer's method with minor modifications [69]. Samples were mixed with 1 ml of solvent mixture (MeOH : CHCl_3_ : H_2_O = 10 : 4 : 4 v/v/v) containing phosphatidylcholine (PC) 15 : 0–18 : 1 (d_7_) (2.1 µM), phosphatidylethanolamine (PE) 15 : 0–18 : 1 (d_7_) (0.07 µM), phosphatidylserine (PS) 15 : 0–18 : 1 (d_7_) (0.064 µM), phosphatidylglycerol (PG) 15 : 0–18 : 1 (d_7_) (0.39 µM), phosphatidylinositol (PI) 15 : 0–18 : 1 (d_7_) (0.12 µM), phosphatidic acid (PA) 15 : 0–18 : 1 (d_7_) (0.10 µM), lysophosphatidylcholine (LPC)18 : 1 (d_7_) (0.47 µM), lysophosphatidylethanolamine (LPE) 18 : 1 (d_7_) (0.10 µM), cholesteryl ester (CE) 18 : 1 (d_7_) (5.3 µM), monoacylglycerol (MG) 18 : 1 (d_7_) (0.055 µM), diacylglycerol (DG) 15 : 0–18 : 1 (d_7_) (0.17 µM), triacylglycerol (TG) 15 : 0–18 : 1 (d_7_) (0.68 µM), sphingomyelin (SM) d18 : 1–18 : 1 (d_9_) (0.41 µM), cholesterol (d_7_) (2.5 µM), ceramide (Cer) d18 : 1–17 : 0 (0.50 µM), hexosylceramide (HexCer) d18 : 1–12 : 0 (0.50 µM), FFA 17 : 0 (0.50 µM), and piperazine-1,4-bis(2-ethanesulfonic acid) (PIPES) (0.63 µM) as internal standards for mass spectrometry-based metabolome analysis. These tubes were vortexed for 60 s, which were subjected to ultrasonic waves for extraction and centrifuged (16 000 × g, 4 °C, 5 min) to remove impurities and foreign substances. Then, 700 µL of the supernatant was transferred to 2 ml Eppendorf tube. After adding 155 µL of H_2_O and 235 µL of CHCl_3_, phase separation of aqueous and organic layers was performed by centrifugation (16 000×**g**, 4°C, 3 min). The aqueous (upper) layer (400 µL) was transferred to a clean tube for analysis by an ion chromatography with a Dionex IonPac AS11-HC-4 µm column (2 mm i.d.    250 mm, 4 µm particle size, Thermo Fisher Scientific, Waltham, MA, U.S.A.) coupled with a quadrupole-Orbitrap mass spectrometry (IC/MS) (Thermo Fisher Scientific) for anionic polar metabolites (i.e. organic acids, nucleotides, etc.) [70,71] and a liquid chromatography with a Discovery HS F5 column (2.1 mm i.d.    150 mm, 3 µm particle size, Merck, Darmstadt, Germany) coupled with a quadrupole-Orbitrap mass spectrometry (PFPP-LC/MS) (Thermo Fisher Scientific) for cationic polar metabolites (i.e. amino acids, etc.) [70,71]. After the aqueous layer extracts were evaporated under vacuum, dried extracts were stored at −80°C until use for IC/MS and PFPP-LC/MS analysis. The organic (lower) layer (200 µL) obtained by phase separation. Finally, 400 µL of MeOH was added and stored at −80°C until analysis. The levels of each hydrophobic metabolites (i.e. TG and CE) were quantified using supercritical fluid chromatography with an ACQUITY UPC^2^ HSS C18 column (3.0 mm i.d.    100 mm, 1.8 µm particle size, Waters, Milford, MA) coupled with a triple quadrupole mass spectrometry (C18-SFC/MS/MS) in multiple reactions monitoring (MRM) mode; the SFC/MS/MS system comprised a SFC (Nexera UC; Shimadzu, Kyoto, Japan) and a triple quadrupole mass spectrometer (LCMS-8060; Shimadzu) [72]. The levels of other lipids (i.e. PC, PE, PS, PG, PI, PA, LPC, LPE, MG, DG, SM, cholesterol, Cer, HexCer, and FFA) were quantified using SFC with an ACQUITY UPC2 Torus diethylamine (DEA) (3.0 mm i.d.    100 mm, 1.8 µm particle size, Waters) coupled with a triple quadrupole mass spectrometry (DEA-SFC/MS/MS) in MRM mode [73]. Supplementary Tables S4–S9 list the abbreviations of the hydrophilic and hydrophobic metabolites.

### DNA microarray analysis

According to the manufacturer's instructions, the cRNA was amplified and labeled using Low Input Quick Amp Labeling, and hybridized using SurePrint G3 Mouse Gene Expression Microarray 8 × 60 K v2 (Agilent Technologies, Santa Clara, CA, U.S.A.). All hybridized microarray slides were scanned using an Agilent scanner. Relative hybridization intensities and background hybridization values were calculated using Agilent Feature Extraction Software (ver. 9.5.1.1).

## Statistical Analysis

All data were expressed as the means ± SEM. Statistical analysis was performed with Student's *t*-test or one-way ANOVA with Fisher's protected least significant difference test. *P* < 0.05 was considered statistically significant.

## Data Availability

Full data will be provided upon reasonable request.

## Abbreviations

ACO: acyl-CoA oxidase
ALT: alanine aminotransferase
ATGL: adipose triglyceride lipase
CANA: canagliflozin
EDL: extensor digitorum longus
eWAT: epididymal white adipose tissue
F6P: fructose 6-phosphate
Fast muscle: fast skeletal muscle
FBP: fructose 1,6-bisphosphate
FFA: free fatty acids
FoxO1: forkhead box transcription factor O1
G6P: glucose-6-phosphate
GS: glutamine synthetase
HK2: hexokinase 2
HOMA-IR: homeostasis model assessment of insulin resistance
HSL: hormone-sensitive lipase
LAT2: L-type amino acid transporter 2
LC3B: microtubule-associated protein 1 light chain 3 β
MCAD: acetyl-CoA dehydrogenase, medium chain
MuRF1: muscle RING-finger protein-1
MyHC: myosin heavy chain
NASH: nonalcoholic steatohepatitis
PDHA1: pyruvate dehydrogenase E1 subunit α 1
PEP: phosphoenolpyruvic acid
PFKm: phosphofructokinase, muscle
PGC1α: peroxisome proliferator-activated receptor α co-activator 1α
PI3K: phosphatidylinositol 3’-kinase
PKm1: pyruvate kinase muscle isozyme 1
PPARA: peroxisome proliferator-activated receptor α
SDH: succinate dehydrogenase
SGLT2: sodium–glucose cotransporter 2
SOL: soleus
slow muscle: slow skeletal muscle
TA: tibialis anterior
β-OHB: β-hydroxybutyric acid
