# Supplementary material for: Differential effect of canagliflozin, a sodium–glucose cotransporter 2 (SGLT2) inhibitor, on slow and fast skeletal muscles from nondiabetic mice
Source: Biochem J. 2022 Feb 11;479(3):425–44. doi: 10.1042/BCJ20210700 (PMC8883489; doi:10.1042/BCJ20210700)
Supplement: Supplementary Figures S1-S10 and Tables S1-S3 [file BCJ-479-425-s1.pdf]

Supplementary Figures:

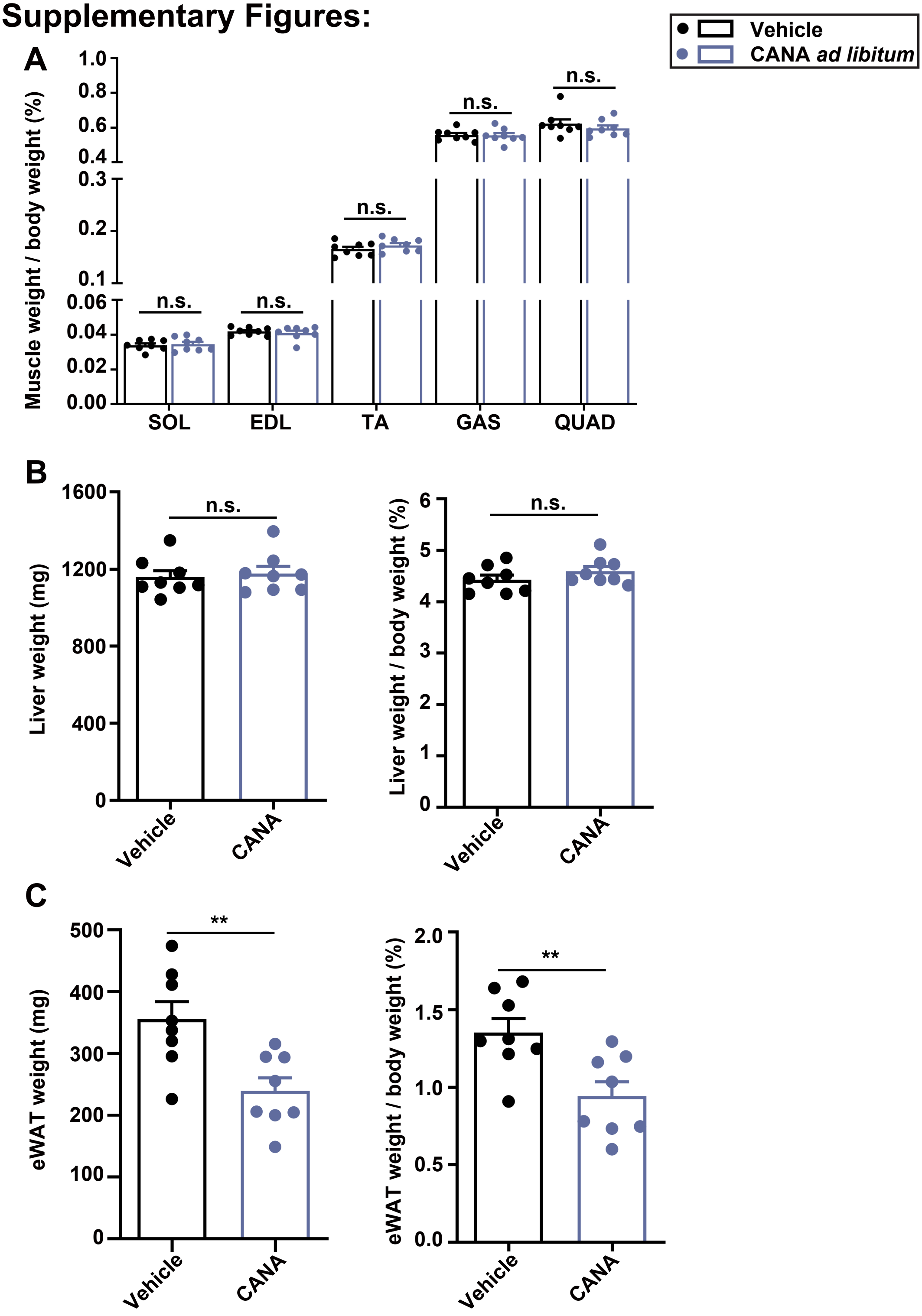

Figure S1. Effect of CANA on tissue weight per body weight during the 2-week *ad libitum* feeding.
(**A**) Right lower limb muscle weight. (**B**) Liver weight. (**C**) eWAT weight. *n* = 8 mice/group.
Data are expressed as the mean ± SEM. ***P* < 0.01 vs. vehicle-treated mice (Student *t*-test).

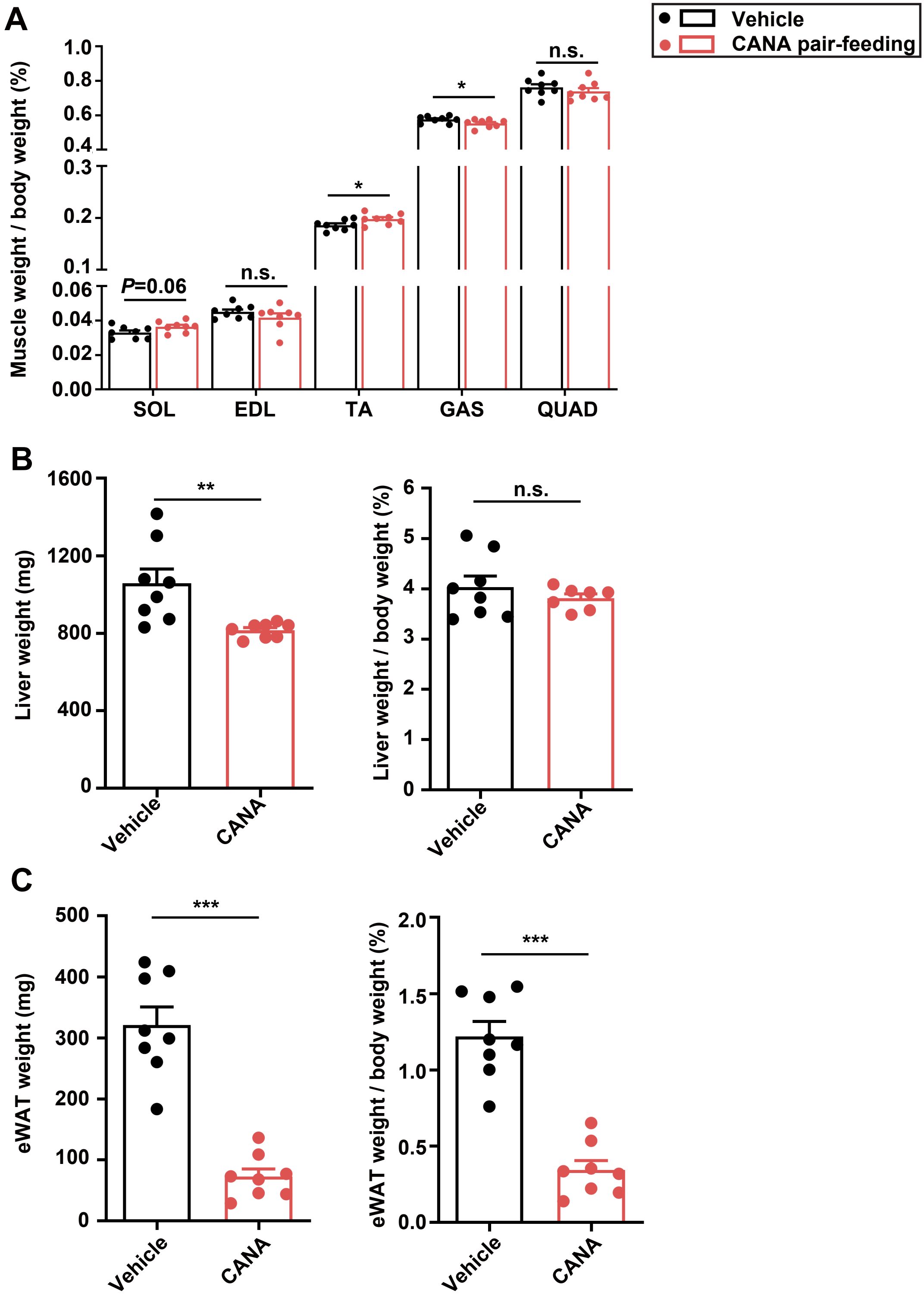

Figure S2. Effect of CANA on tissue weight per body weight during the 2-week pair-feeding.
(**A**) Right lower limb muscle weight. (**B**) Liver weight. (**C**) eWAT weight. n = 8 mice/group.
Data are expressed as the mean ± SEM. *$P < 0.05$, **$P < 0.01$, ***$P < 0.001$ vs. vehicle-treated mice (Student t-test).

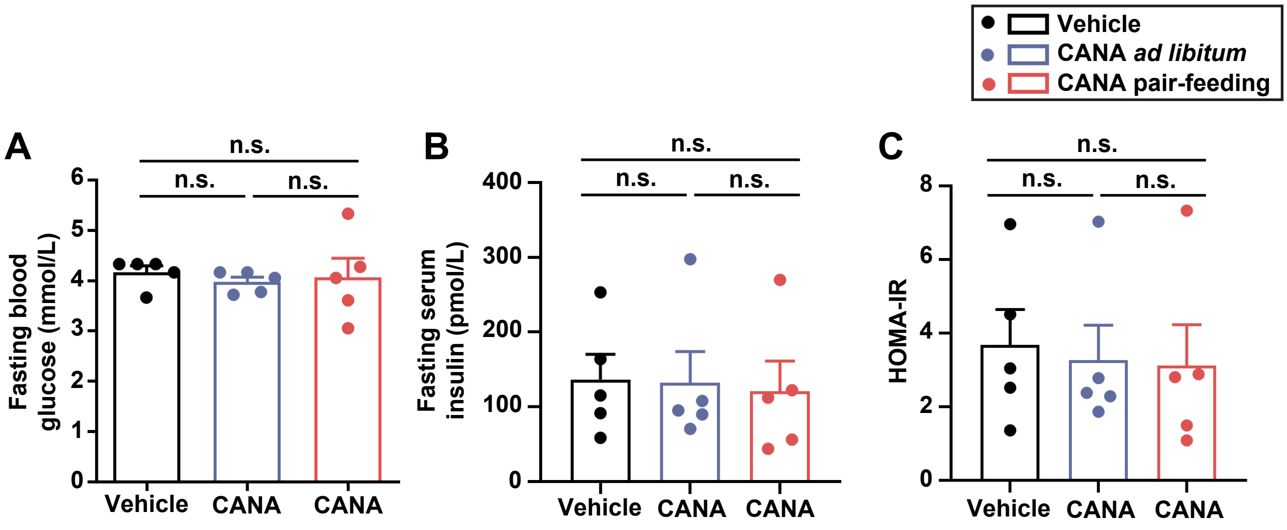

Figure S3. Effect of CANA on blood glucose, serum insulin and HOMA-IR after 16 hours fasting during the 4-week *ad libitum* or pair-feeding.

Mice were divided into 3 groups; those treated with vehicle and those with CANA during the 4-week *ad libitum* feeding, and those treated with CANA during the 4-week pair-feeding.

(**A**) Fasting blood glucose. (**B**) Fasting serum insulin. (**C**) HOMA-IR. n = 5 mice/group. Data are expressed as the mean ± SEM. n.s. = not significant (ANOVA).

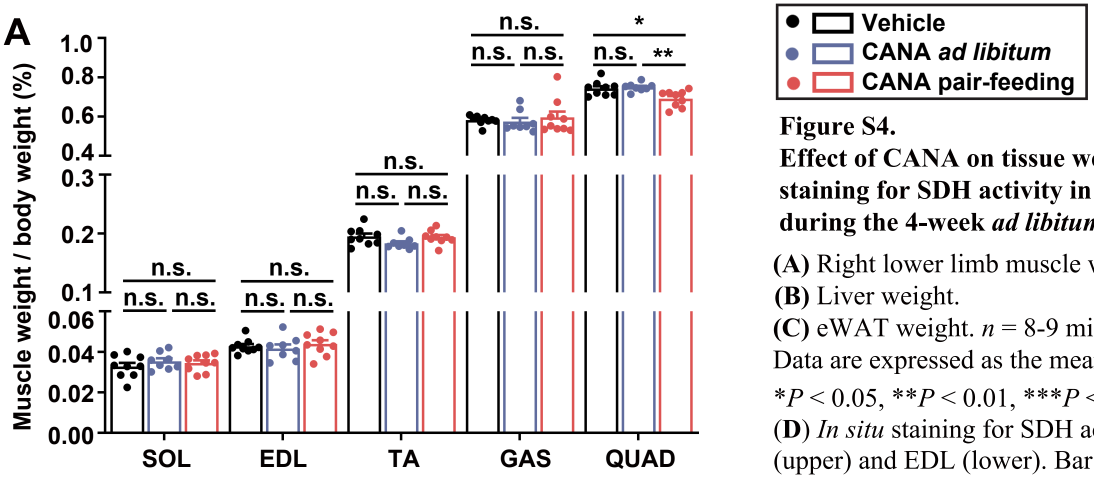

Figure S4.
Effect of CANA on tissue weight and staining for SDH activity in the muscles during the 4-week *ad libitum* or pair-feeding.

(A) Right lower limb muscle weight.
(B) Liver weight.
(C) eWAT weight. n = 8-9 mice/group.
Data are expressed as the mean ± SEM.
**P* < 0.05, ***P* < 0.01, ****P* < 0.001 (ANOVA).
(D) *In situ* staining for SDH activity in SOL (upper) and EDL (lower). Bar = 100 μm.
n = 5 mice/group. Data are expressed as the mean ± SEM. ***P* < 0.01 (ANOVA).

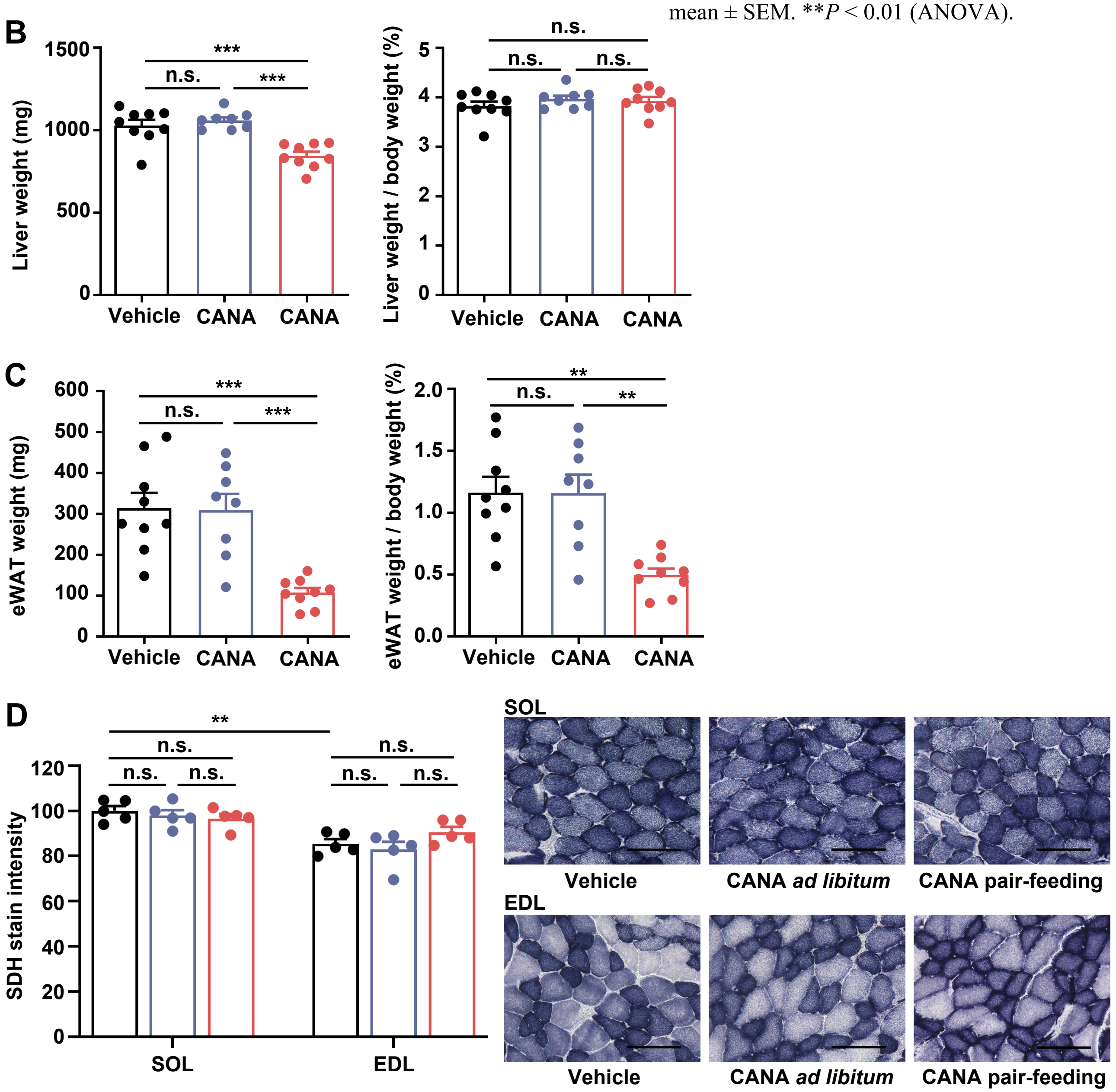

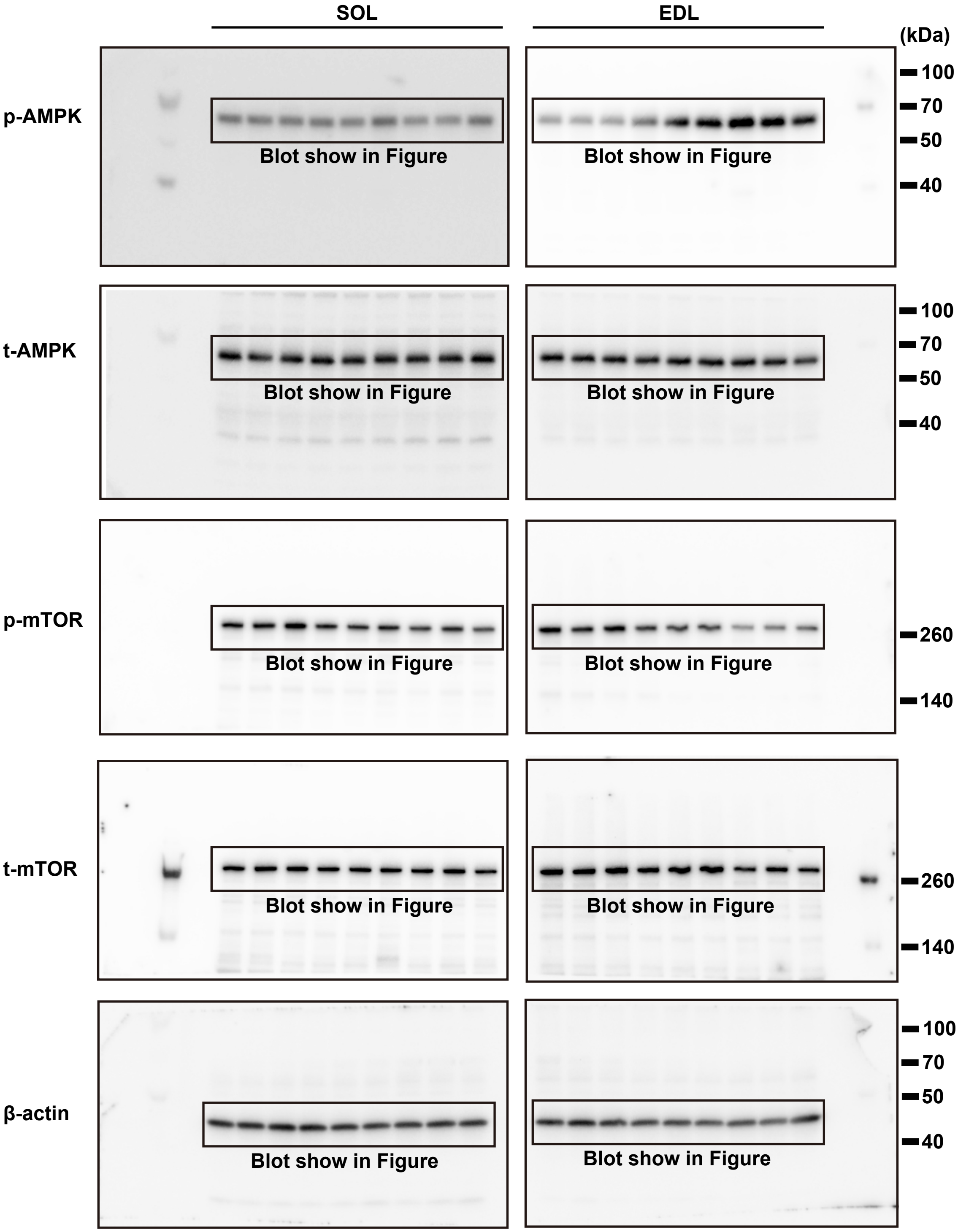

Figure S5. Entire unmodified western blots for Figure 6A

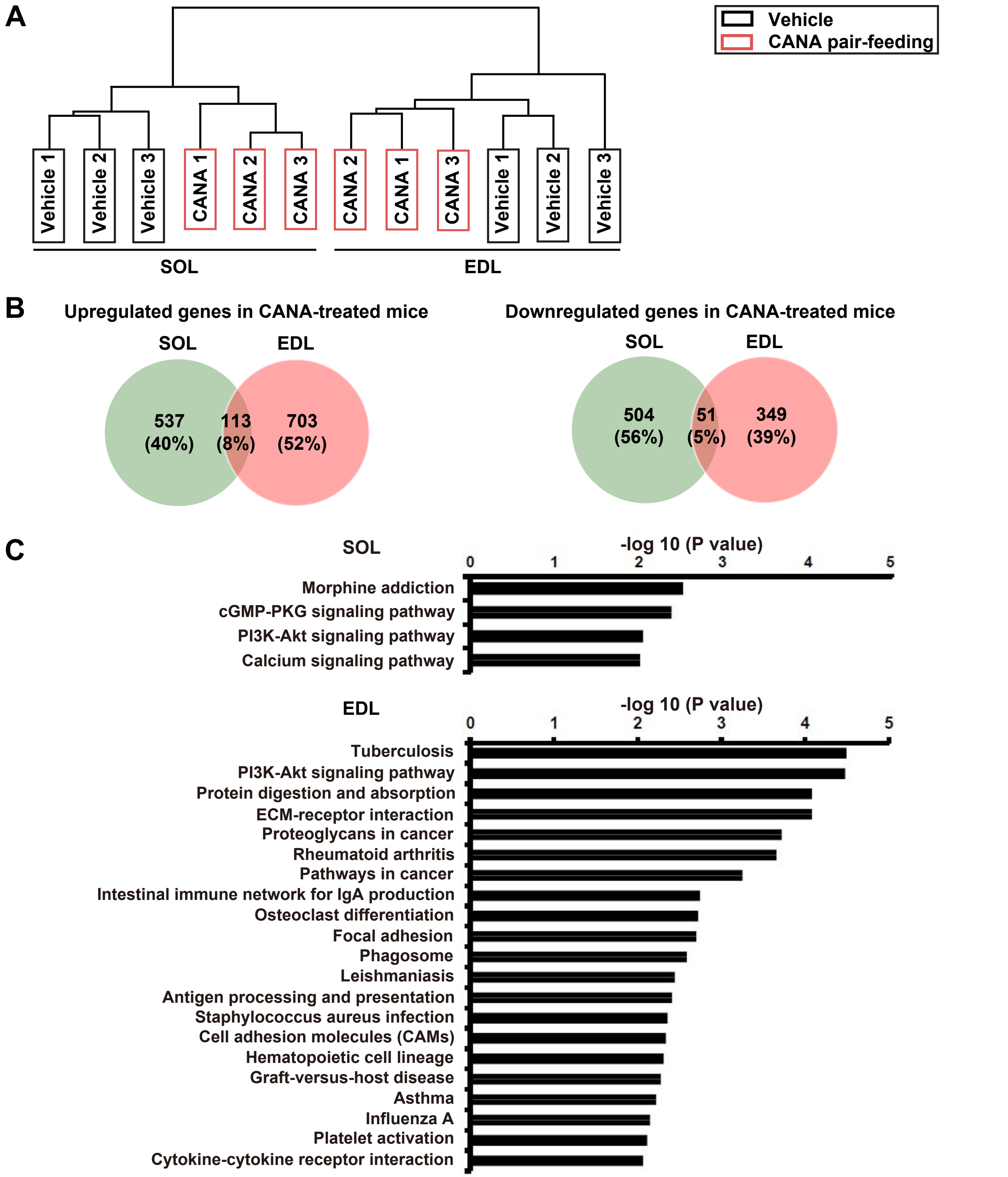

Figure S6. Microarray analysis of SOL and EDL from mice treated with vehicle and those treated with CANA during the 4-week pair-feeding.

(**A**) Hierarchical clustering by using the signal data of the microarray datasets of SOL and EDL from CANA- and vehicle-treated mice (n = 3 mice/group: CANA 1-3 and Vehicle 1-3 for CANA- and vehicle-treated mice, respectively). (**B**) Venn diagrams of upregulated and downregulated genes in SOL and EDL by CANA. (**C**) KEGG pathway functional classification of genes differentially expressed in SOL and EDL after CANA treatment. DAVID v6.8 functional annotation bioinformatics microarray analysis software was used to obtain the KEGG pathway functional classification. KEGG pathway terms for classification that showed statistically significantly differences in the amount of genes (compared with vehicle) are shown ($P \leq 0.01$).

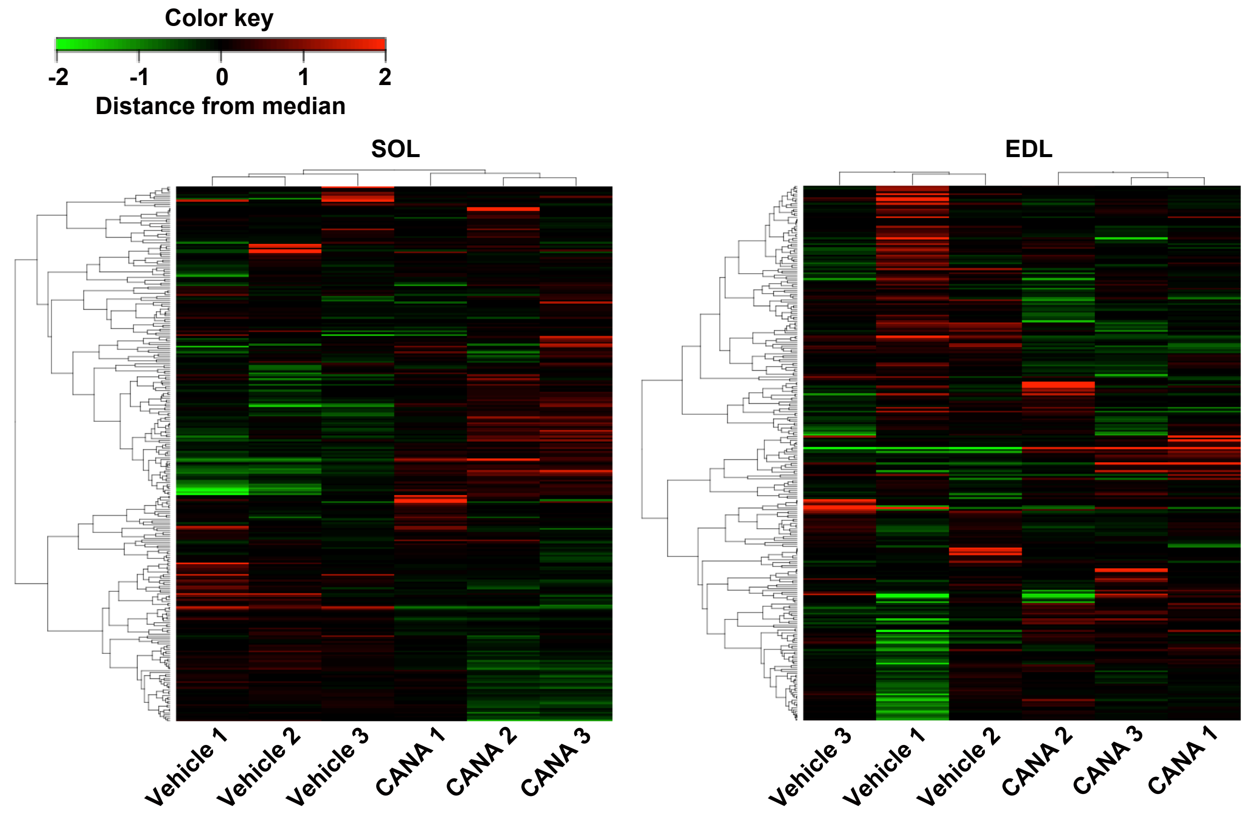

Figure S7. Hierarchical clustering in PI3K-Akt signaling pathway during the 4-week pair-feeding. The heat map generated by MeV software. Hierarchical clustering by using the signal data of the microarray datasets of SOL and EDL from CANA- and vehicle-treated mice during the 4-week pair-feeding (*n* = 3 mice/group: CANA 1-3 and Vehicle 1-3 for CANA- and vehicle-treated mice, respectively). The color indicates the distance from the median of each row (The distance metric is “Pearson Correlation”, and the linkage method is “average linkage clustering”).

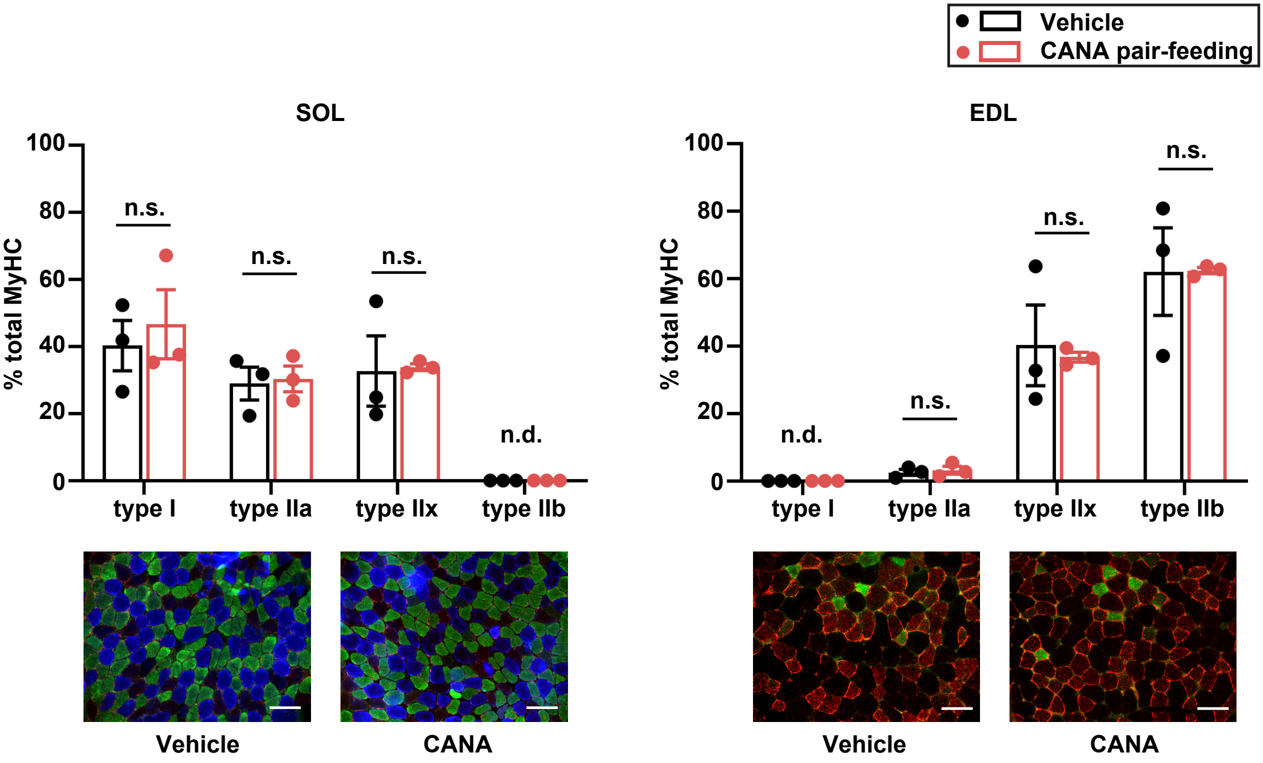

Figure S8. *In situ* immunolabeling of SOL and EDL during the 4-week pair-feeding.
In situ immunolabeling of a muscle cross-section for MyHC type I (blue), type IIa (green), type IIx (black) and type IIb (red). Bar = 100 µm. n = 3 mice/group. Data are expressed as the mean ± SEM. (Student t-test).

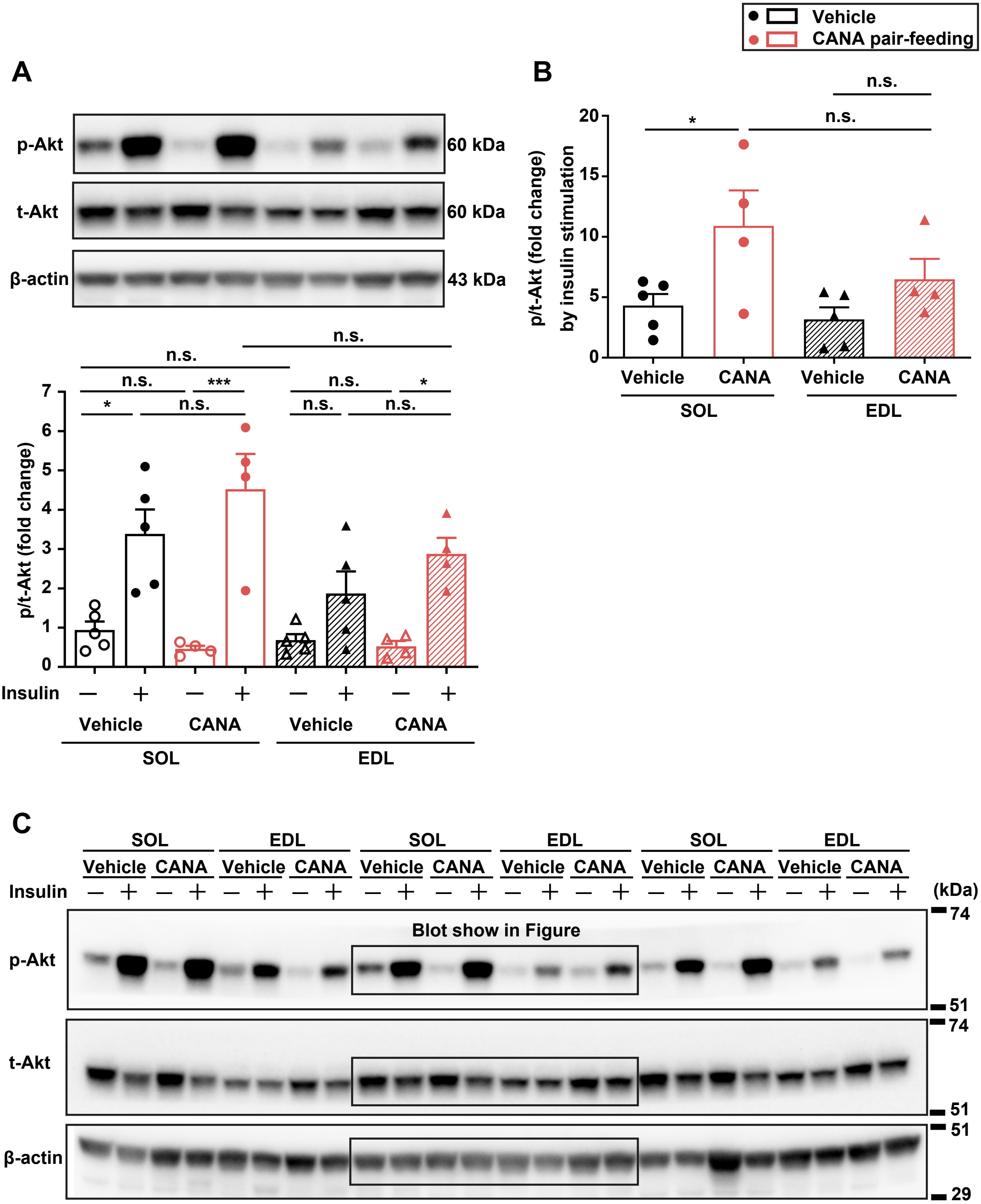

Figure S9. Effect of CANA on the insulin-stimulated p-Akt in SOL and EDL during the 4-week pair-feeding. (A) Representative Western blots for the assessment of p-Akt (upper) and quantitative bar graphs (lower) are shown. **(B)** Insulin-stimulated p-Akt in SOL and EDL. $n = 4$-5 mice/group. Data are expressed as mean ± SEM. $^*P < 0.05$, $^{***}P < 0.001$ (ANOVA). **(C)** Entire unmodified western blots.

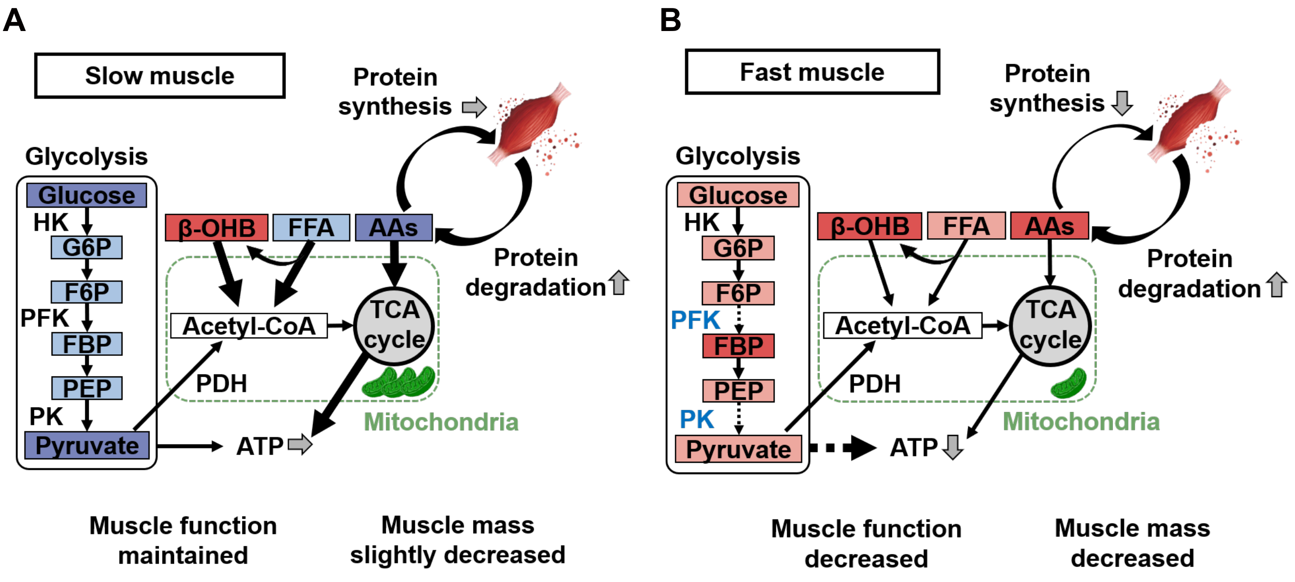

Figure S10. Schematic illustration of the differential effect of SGLT2 inhibition on slow muscle (A) and fast muscle (B) during the 4-week pair-feeding.

Key metabolites and metabolic enzymes are shown. The colors for metabolites are described in Figure 2. As for the colors of metabolic enzymes, blue and black indicate those which are decreased and unaffected, respectively, in CANA-treated mice relative to vehicle-treated mice.

Supplementary Tables:

Table S1 The primer sequences list of the selected genes for quantitative Real-time PCR

Genes	Forward primers（5'-3'）	Reverse primers（5'-3'）
Foxo1	GCGGGCTGGAAGAATTCAAT	TCCAGTTCCTTCATTCTGCA
Atrogin1	ATGCCGTTCCTTGGGCAGGA	TCAGAACTTGAACAAATTGA
Murf1	ATGAACTTCACGGTGGGTTT	TCAGTGCAGGCCTGAGCCTT
Lc3b	CGATACAAGGGGGAGAAGCA	ACTTCGGAGATGGGAGTGGA
P62	TGCTCTTCGGAAGTCAGCAA	CCCGACTCCATCTGTTCCTC
Glut4	AAAAGTGCCTGAAACCAGAG	TCACCTCCTGCTCTAAAAGG
Lat2	AAGAAGCCTGACATTCCCCG	TGTGTTGCCAGTAGACACCC
Cd36/fat	TCCTCTGACATTTGCAGGTCTATC	AAAGGCATTGGCTGGAAGAA
Hk2	CGGTACACTCAATGACATCC	GTAGACAGAGCCATCCACG
Pfkm	GGAGTGCGTGCAGGTGACCAAA	ATCACGGCCACTGTGTGCAACC
Pkm1	GCTGTTTGAAGAGCTTGTGC	TTATAAGAGGCCTCCACGCT
Pdha1	ACATGGCTTCACCTTCACTC	CCGTTGCCTCCATAGAAGTTC
Bcat2	CGGACCCTTCATTCGTCAGA	CCATAGTTCCCCCCCAACTT
Bckdha	CCAGGGTTGGTGGGATGAG	GGCTTCCATGACCTTCTTTCG
Bckdk	GATCCGAATGCTGGCTACTCA	GCCAACAAAATCAGGCTTGTC
Alt1	GCGCCAGGGTGTGAAGAA	GCTTGTGCATCCCCAATATTG

Alt2	GAAGGAAGTAGCCGCATCCA	AGGAAAAGCTGTAGACCATCACA
Gs	GCTGCAAGACCCGTACCCT	TTCCACTCAGGTAACTCTTCCACA
Aco	GGCCAACTATGGTGGACATCA	ACCAATCTGGCTGCACGAA
Mcad	TAATCGGTGAAGGAGCAGGTTT	GGCATACTTCGTGGCTTCGT
Cpt1a	CCTGGGCATGATTGCAAAG	GGACGCCACTCACGATGTT
Ppara	CTGCAGAGCAACCATCCAGAT	GCCGAAGGTCCACCATTTT
Atgl	ATTTATCCCGGTGTACTGTG	GGGACACTGTGATGGTATTC
Hsl	ACTCAGACCAGAAGGCACTA	TAGTTCCAGGAAGGAGTTGA
Pgc1a	TATGGAGTGACATAGAGTGTGCT	CTGGGCAAAGAGGCTGGTC
18s	GCTTAATTTGACTCAACACGGGA	AGCTATCAATCTGTCAATCCTGTC

Table S2 Antibodies used for immunolabeling

**Primary antibody cocktails and concentrations**	**MyHC reactivity**	**Providers**	**Secondary antibody cocktails and concentraion**	**Providers**
**BA-F8 (1:25)**	**I**	**DSHB**	**Alexa Fluor 350 IgG2b (1:1000)**	**Invitrogen**
**SC-71 (1:40)**	**IIa**	**DSHB**	**Alexa Fluor 488 IgG1 (1:2000)**	**Invitrogen**
**BF-F3 (1:40)**	**IIb**	**DSHB**	**Alexa Fluor 555 IgM (1:1000)**	**Invitrogen**

Table S3 Antibodies used for Western blot analysis

**Target proteins**	**Hosts**	**Providers**	**Catalog No.**	**Dilution**
Phospho-AMPKα (Thr172) (40H9)	rabbit	Cell Signaling Technology	2535	1:1000
AMPKα	rabbit	Cell Signaling Technology	2532	1:1000
Phospho-mTOR (Ser2448)	rabbit	Cell Signaling Technology	2971	1:1000
mTOR Antibody	rabbit	Cell Signaling Technology	2972	1:1000
Phospho-Akt (Ser473) Antibody	rabbit	Cell Signaling Technology	9271	1:1000
Akt Antibody	rabbit	Cell Signaling Technology	9272	1:1000
Anti-β-Actin Antibody (1-19), HRP Conjugate	goat	Santa Cruz Biotechnology	sc-1616	1:5000
Anti-rabbit IgG, HRP-linked Antibody	donkey	Jackson Immuno Research	711-036-152	1:10000
